# Stepwise Maturation of Lytic Granules during Differentiation and Activation of Human CD8^+^ T Lymphocytes

**DOI:** 10.1371/journal.pone.0027057

**Published:** 2011-11-04

**Authors:** Yovan Sanchez-Ruiz, Salvatore Valitutti, Loic Dupre

**Affiliations:** 1 INSERM, U1043, Toulouse, France; 2 CNRS, U5282, Toulouse, France; 3 Université Toulouse III Paul-Sabatier, Centre de Physiopathologie de Toulouse Purpan, Toulouse, France; Ohio State University Medical Center, United States of America

## Abstract

During differentiation, cytotoxic T lymphocytes (CTL) acquire their killing potential through the biogenesis and maturation of lytic granules that are secreted upon target cell recognition. How lytic granule load in lytic molecules evolves during CTL differentiation and which subsets of lytic granules are secreted following activation remains to be investigated. We set up a flow cytometry approach to analyze single lytic granules isolated from primary human CTL according to their size and molecular content. During CTL in vitro differentiation, a relatively homogeneous population of lytic granules appeared through the progressive loading of Granzyme B, Perforin and Granzyme A within LAMP1^+^ lysosomes. PMA/ionomycin-induced lytic granule exocytosis was preceded by a rapid association of the docking molecule Rab27a to approximately half of the lytic granules. Activated CTL were found to limit exocytosis by sparing lytic granules including some associated to Rab27a. Our study provides a quantification of key steps of lytic granule biogenesis and highlights the potential of flow cytometry to study organelle composition and dynamics.

## Introduction

Lytic granules play a crucial role in CD8^+^ cytotoxic T lymphocytes (CTL)-mediated killing of infected and transformed cells [1]–[3]. Following antigenic challenge, efficient immune responses are associated with the ability of activated CD8^+^ T lymphocytes to differentiate into effector and memory cells [4], [5]. During this differentiation process, lytic molecules such as Granzyme A (GrA), Granzyme B (GrB) and Perforin (Pfp) are expressed and stored for secretion into lytic granules [6]–[8].

Lytic granules have been referred to as secretory lysosomes since they act as dual-functional organelles, carrying out both secretory and degradative functions [9], [10]. As relatives of lysosomes, lytic granules have low intra-luminal pH, contain lysosomal hydrolases such as cathepsins and harbor the lysosomal associated membrane proteins LAMP1, LAMP2 and LAMP3 [8], [11]. In addition, lytic granules share features with endosomes since they incorporate extra-cellular proteins and surface receptors such as the TCR, CD8 and MHC class I [12]. Electron microscopy (EM) studies have shown that lytic granules have a diameter ranging from 0.5 to 2 µm. They are membrane-delimited and present variable content in multivesicular regions and dense cores [7], [8], [13]. This morphological heterogeneity may correspond to sequential intermediates on the endocytic pathway, in a context in which late endosomes and secretory lysosomes fuse with each other [14].

The dynamics of lytic granule contents during CTL differentiation and following CTL activation have previously not been fully characterized. Murine T cell activation leads to an extensive architectural reorganization of the lysosomal compartment with the transition from few LAMP1^+^ small cytoplasmic vesicles to numerous large LAMP1^+^ vesicular structures [6]. Human CTL clone activation leads to the progressive association of GrA to LAMP1-2^+^ organelles, suggesting that lytic molecules accumulate within pre-existing or newly formed lysosomes [14]. Rapidly after an effector CTL encounters a target cell presenting cognate antigenic peptides, lytic granules are delivered into the intercellular cleft [15], which correlates with the appearance of LAMP1 at the CTL surface and the concurrent loss of intracellular lytic molecules [16], [17]. It is considered that lytic granules are not intrinsically prone to secretion, since their exocytosis requires the acquisition of molecules promoting membrane docking and fusion, such as Rab27a and Munc14-3, via the fusion with endosomal/exocytic vesicles [18]–[20].

Most studies on lytic granules are based on confocal and electron microscopy. Therefore a quantitative assessment of lytic granule composition and load during their biogenesis and maturation is still missing. Here, we developed a flow cytometry approach to characterize the composition of individual lytic granules deriving from primary human CTL. Our study indicates that during CTL differentiation, lytic granules arise from the stepwise loading of lytic molecules into lysosomes. Our approach furthermore reveals that, following activation, differentiated CTL recruit Rab27a^+^ only on a set of lytic granules and that they do not secrete all of them.

## Results

### Rise in lytic molecule load during primary human CD8^+^ T cell differentiation

**Figure 1:**
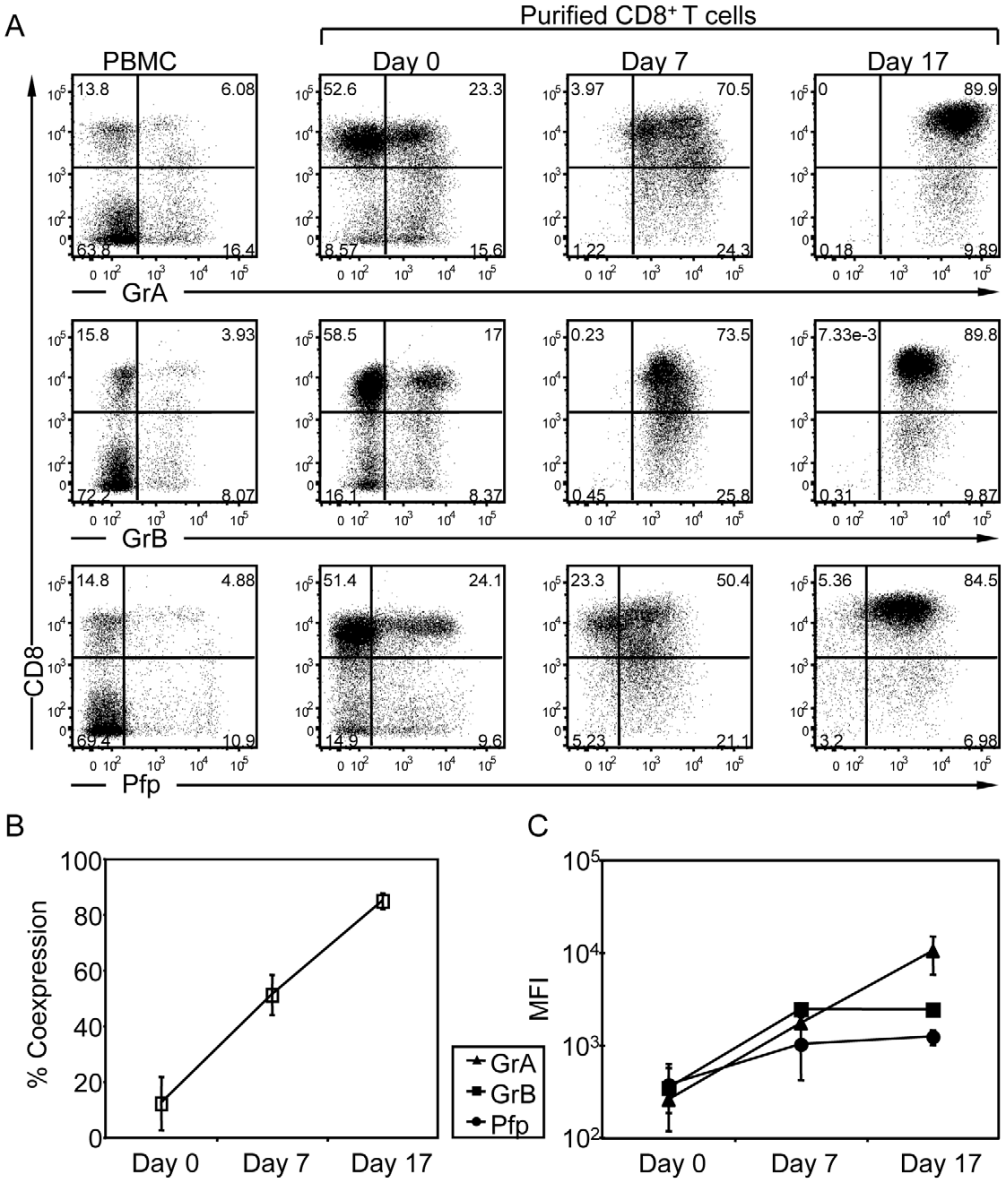
Expression of lytic molecules during the in vitro expansion of primary CD8^+^ T cells. (A) Expression of GrA, GrB and Pfp in human PBMC and purified CD8^+^ T cells at days 0, 7 and 17 after stimulation with anti-CD3/CD28 mAb-coated beads in the presence of IL-2, Il-7 and IL-15. Cells were stained with anti-CD8, anti-GrA, anti-GrB and anti-Pfp mAbs and analyzed by flow cytometry. The expression levels of GrA, GrB and Pfp are plotted against the CD8 marker. The values in each plot show the frequency of each subset in the cell population. The data are from one representative donor out of 3 analyzed in a similar way. (B) Frequency of the cells expressing the three lytic molecules GrA, GrB and Pfp. Percentage of co-expression was measured by first gating the CD8 and GrA positive cells and then on the GrB and Pfp positive cells. The mean percentage and SD of the resulting population at days 0, 7 and 17 are shown (n = 3). (C) The mean fluorescence intensity (MFI) of GrA, GrB and Pfp for CD8^+^ cells at days 0, 7 and 17 are shown (n = 3).

To obtain primary human CTL, CD8^+^ T cells were purified from PBMC and expanded with anti-CD3/CD28 mAb-coated beads supplemented with a cytokine cocktail. As expected, PBMC contained a minor fraction of CD8^+^ T cells expressing the lytic molecules GrA, GrB and Pfp (**Fig. 1A**). Upon negative selection using magnetic beads, a cell population highly enriched in CD8^+^ T cells was obtained (Day 0). The fraction of purified CD8^+^ T cells displaying an effector phenotype on the basis of the co-expression of GrA, GrB and Pfp was about 15% (**Fig. 1B** **and Fig. S1**). Following anti-CD3/CD28 mAb-driven stimulation, the percentage of CD8^+^ T cells expressing GrA, GrB and Pfp progressively increased (**Fig. 1A–B**). At day 7 after stimulation, a majority of CD8^+^ T cells expressed the lytic molecules, although at heterogeneous levels. Approximately 70% of the CTL expressed GrA and GrB, while only 50% of them expressed Pfp. At day 17 after stimulation, the majority (>80%) of CD8^+^ T cells expressed all 3 lytic molecules at homogeneous levels (**Fig. 1A–B**). Similarly high levels of lytic molecule expression were measured in a 14- to 20-day differentiation window. Analysis of the mean fluorescence intensity indicates that the levels of the 3 lytic molecules per cell basis increased along differentiation (**Fig. 1C**). These data show that following CD8^+^ T cell purification and in vitro stimulation, CTL differentiate by gradually acquiring lytic molecules. CTL at intermediate (Day 7) and full (Days 14 to 20) differentiation stages were used for further experiments.

### Heterogeneous morphology of vesicles isolated from primary human CD8^+^ T cells

**Figure 2:**
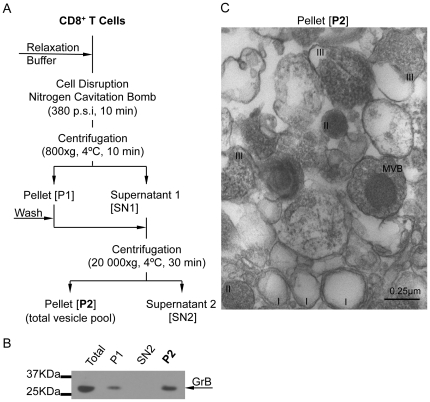
Isolation of lytic granules from CTL. (A) Schematic representation of the simplified procedure used to isolate a vesicular extract enriched in lytic granules. (B) Aliquots corresponding to the CTL homogenate, the P1 pellet, the supernatant SN2 and the P2 pellet were immunoblotted with an antibody specific for GrB. (C) Electron micrograph of the P2 pellet fraction (Magnification, 40 000×). Distinct vesicles can be observed: tiny droplets (I), dark core bodies surrounded by lipid bilayers (II), larger granules containing small internal vesicles (III), granules containing multivesicular bodies (MVB).

To isolate vesicles from CD8^+^ T cells, a differential centrifugation strategy was used (**Fig. 2A**). Twenty million differentiated CTL were disrupted in relaxation buffer using a nitrogen cavitation bomb. The cell homogenate was first centrifuged at low-speed to pellet nuclei, membranes, debris and part of mitochondria (P1). This fraction was washed to recover residual vesicles. The supernatant (SN1) was added to the wash of P1 and then submitted to high-speed centrifugation to pellet the vesicles (P2). To evaluate whether this procedure allowed the enrichment in vesicles containing lytic proteins, the total cell homogenate, as well as the fractions P1, SN2 and P2 were examined for GrB content by Western blotting (**Fig. 2B**). The P1 fraction contained residual GrB, validating the use of the wash step. Clearly, the P2 fraction was enriched in GrB, while the SN2 fraction was free of GrB. The analysis of the P2 fraction by transmission electron microscopy (TEM) revealed the presence of vesicle subtypes displaying various morphology and variable size (**Fig. 2C**). In particular, we identified tiny droplets (I), dark-core bodies surrounded by a thin membrane (II), larger granules containing small internal vesicles (III), and multivesicular bodies (MVB). The diameter of these morphologically heterogeneous vesicles appeared to range between 0.1 to 1.3 µm. This analysis indicates that the crude vesicular extract of differentiated CTL obtained by a simple differential centrifugation procedure was enriched in lytic granules on the basis of size, morphology and GrB content.

### Flow cytometry analysis of the size and granulometry of individual CTL vesicles

**Figure 3:**
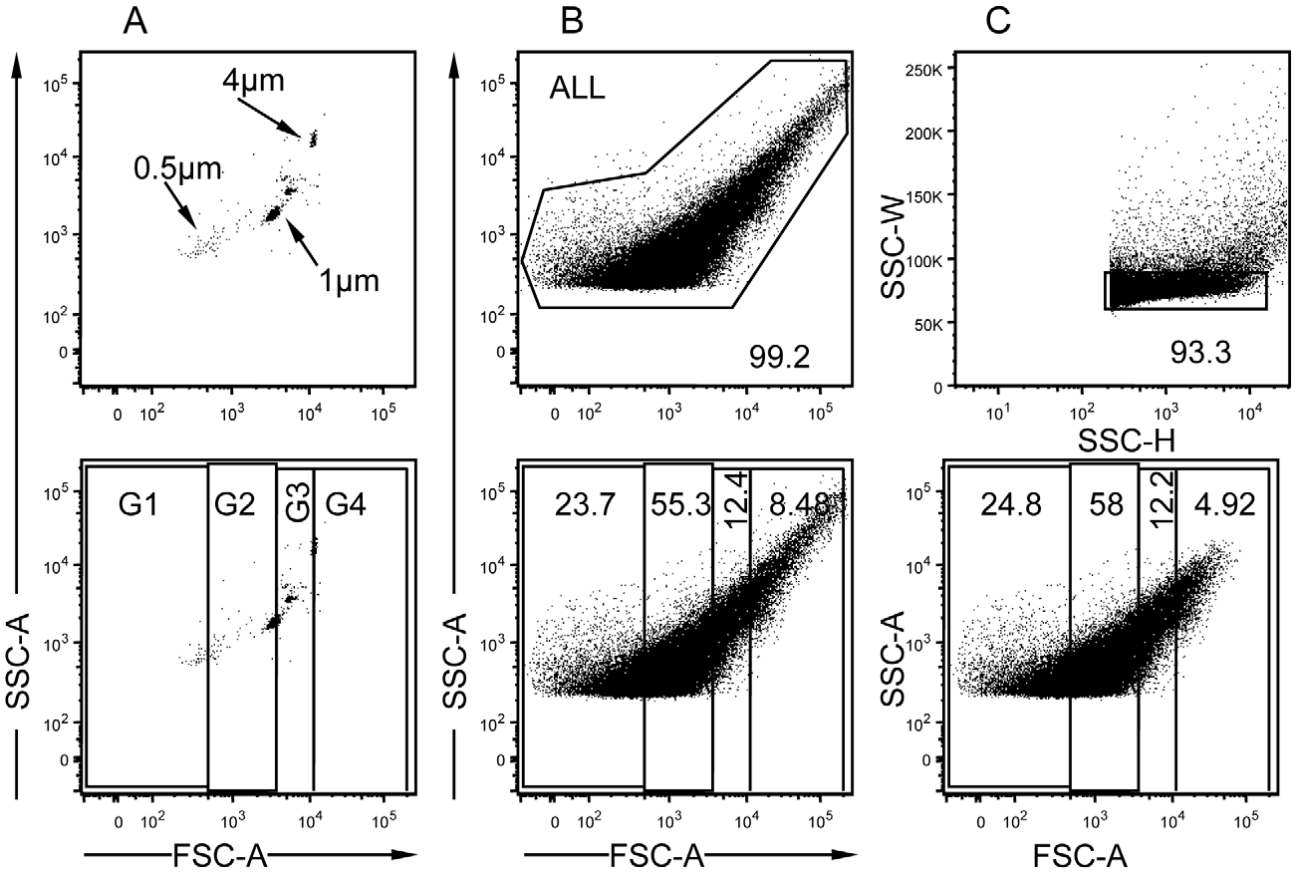
Analysis of CTL vesicle physical parameters by flow cytometry. (A) Upper panel shows size standard beads of 0.5, 1.0 and 4.0 µm used to set-up the SSC and FSC parameters of the flow cytometer. Lower panel shows the design of 4 size-based (FSC) gates (G1 to G4), which borders were placed at the center of distribution of size standard beads. (B) Upper panel shows crude P2 fraction acquired with identical instruments setting used for the size standard beads. The gate “ALL” encompasses the majority of the vesicles. Lower panel shows size based gating analysis of the crude P2 fraction. (C) Doublets were removed by gating on a plot of side-scatter height (SSC-H) versus side-scatter width (SSC-W). Lower panel shows size-based gating analysis of the P2 fraction after the doublet discrimination. Plots corresponding to one representative vesicular extract are shown.

To study the vesicles isolated from human CTL by flow cytometry, latex particles of distinct known sizes (0.5, 1 and 4 µm) were used to set up the flow cytometer FSC and SCC parameters (**Fig. 3A**). On the basis of these standard particles, we established 4 size gates, G1 (<0.5 µm), G2 (0.5 to 1 µm), G3 (1 to 4 µm) and G4 (>4 µm). The flow cytometry analysis of the crude P2 fraction isolated from differentiated CTL showed a population of events with a relatively continuous size and granulometry distribution (**Fig. 3B**). Most of these events distributed either in gates G1 or G2, in comparable proportions (**Fig. 3B** **and Fig. S2**). To eliminate possible debris and aggregates present in the crude P2 fraction, a stringent strategy was applied [21]. Debris and aggregates are expected to have different pulse widths as compared to the pool of single vesicles. Briefly, events displaying high Side-Scatter Width (SSC-W) pulses as compared to the main pool of single vesicles were excluded (**Fig. 3C**, upper panel), which resulted in the disappearance of those events with the highest size (FSC-A, lower panel). This gating strategy was applied to all further vesicle analysis. This flow cytometry approach shows that vesicles isolated from primary human CTL display a wide but fairly continuous size and granulometry distribution.

### Flow cytometry identification of conventional lysosomes and lytic granules

**Figure 4:**
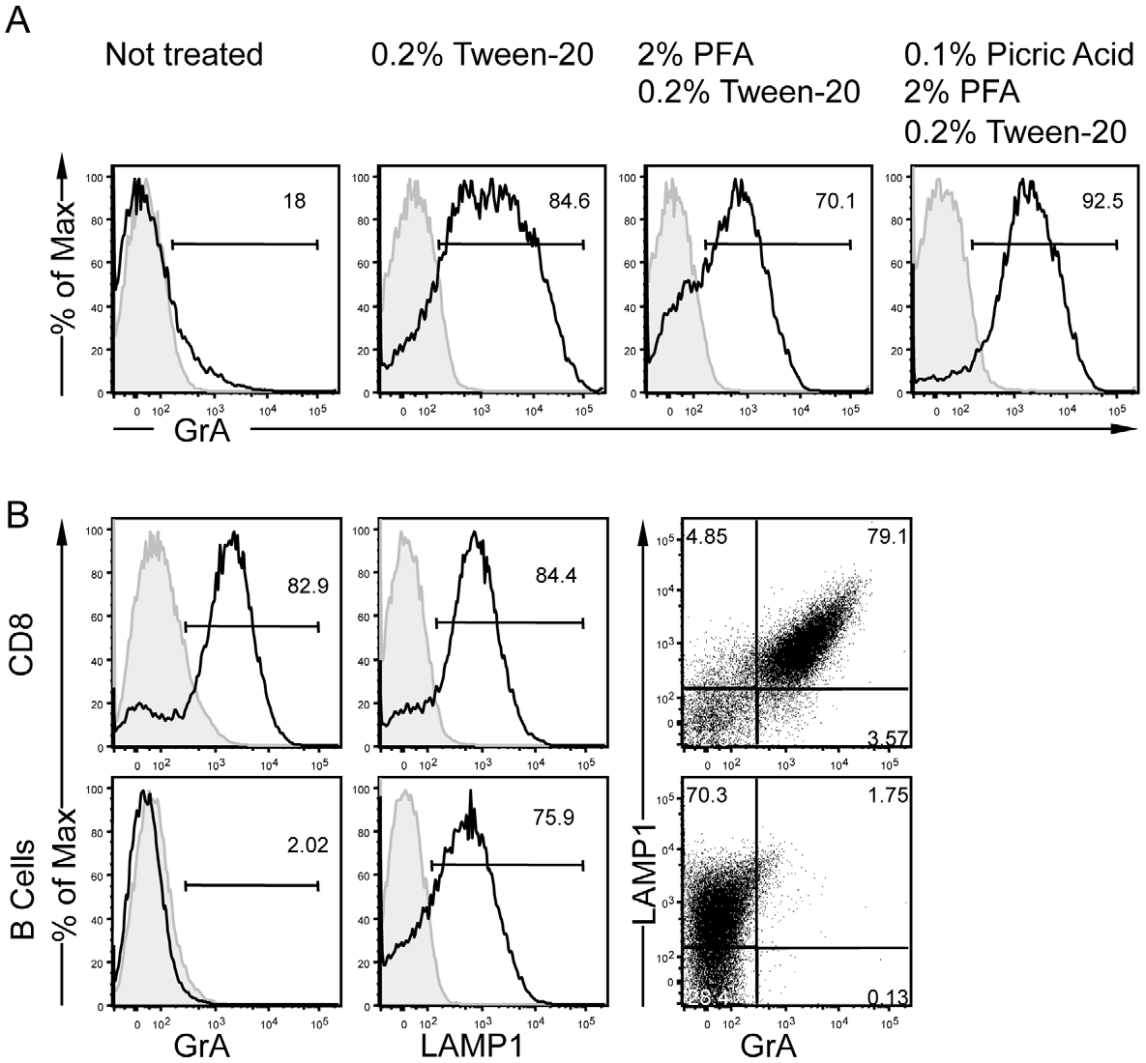
GrA staining discriminates lytic granules from conventional lysosomes. (A) GrA staining of the vesicular extract from differentiated CTL treated with the indicated fixation/permeabilization conditions. (B) Vesicular extracts from CTL (up) and from JY (EBV-transformed B cells) (down) were fixed with 0.2% PFA in the presence of 0.1% picric acid, permeabilized with 0.2% Tween-20 and stained with anti-GrA and anti-LAMP1 mAbs. Represented are also the simultaneous labeling of both subcellular fractions. The data are from one representative experiment out of 3 experiments.

Different conditions to fix/permeabilize isolated vesicles were compared in their ability to allow specific immunolabeling of the intra-granular marker GrA. As expected, unpermeabilized vesicles were not labeled with the GrA fluorescent Ab. In contrast, the different fixation/permeabilization conditions tested allowed a specific staining for GrA (**Fig. 4A**). The condition including 0.1% picric acid, 2% PFA and 0.2% Tween-20 was selected for further experiments since it yielded the highest GrA staining, in agreement with the fact that picric acid is expected to better preserve the nature of epitopes [22]. We then stained vesicles from differentiated CD8^+^ CTL using GrA and LAMP1 fluorescent antibodies simultaneously. Nearly 80% of the CTL vesicles co-expressed these markers (**Fig. 4B**), thereby strongly indicating that most vesicles isolated from CTL were lytic granules. In order to further control the specificity of the GrA immunolabeling, vesicles isolated from B lymphocytes were fixed/permeabilized and incubated with LAMP1 and GrA fluorescent antibodies. Although a majority of vesicles isolated from B lymphocytes was specifically labeled with the LAMP1 fluorescent Ab, these vesicles did not display a specific staining for GrA (**Fig. 4B**). This result validates the specificity of the GrA immunolabeling and indicates that most vesicles isolated from B lymphocytes were conventional lysosomes. To test whether the lack of detection of lysosomes (LAMP1^+^/GrA^−^) in CTL could result from an artefactual fusion between lytic granules and lysosomes, we mixed, prior to the immunolabeling, vesicles purified from B lymphocytes and CTL. Each vesicle population remained distinct (**Fig. S3**), thereby indicating that no major fusion between lytic granules and lysosomes occurred during the in vitro manipulation. Together, our data demonstrate that immunolabeling followed by flow cytometry analysis is applicable to the study of conventional lysosomes and lytic granules.

### Transition from conventional lysosomes to lytic granules during CTL differentiation

**Figure 5:**
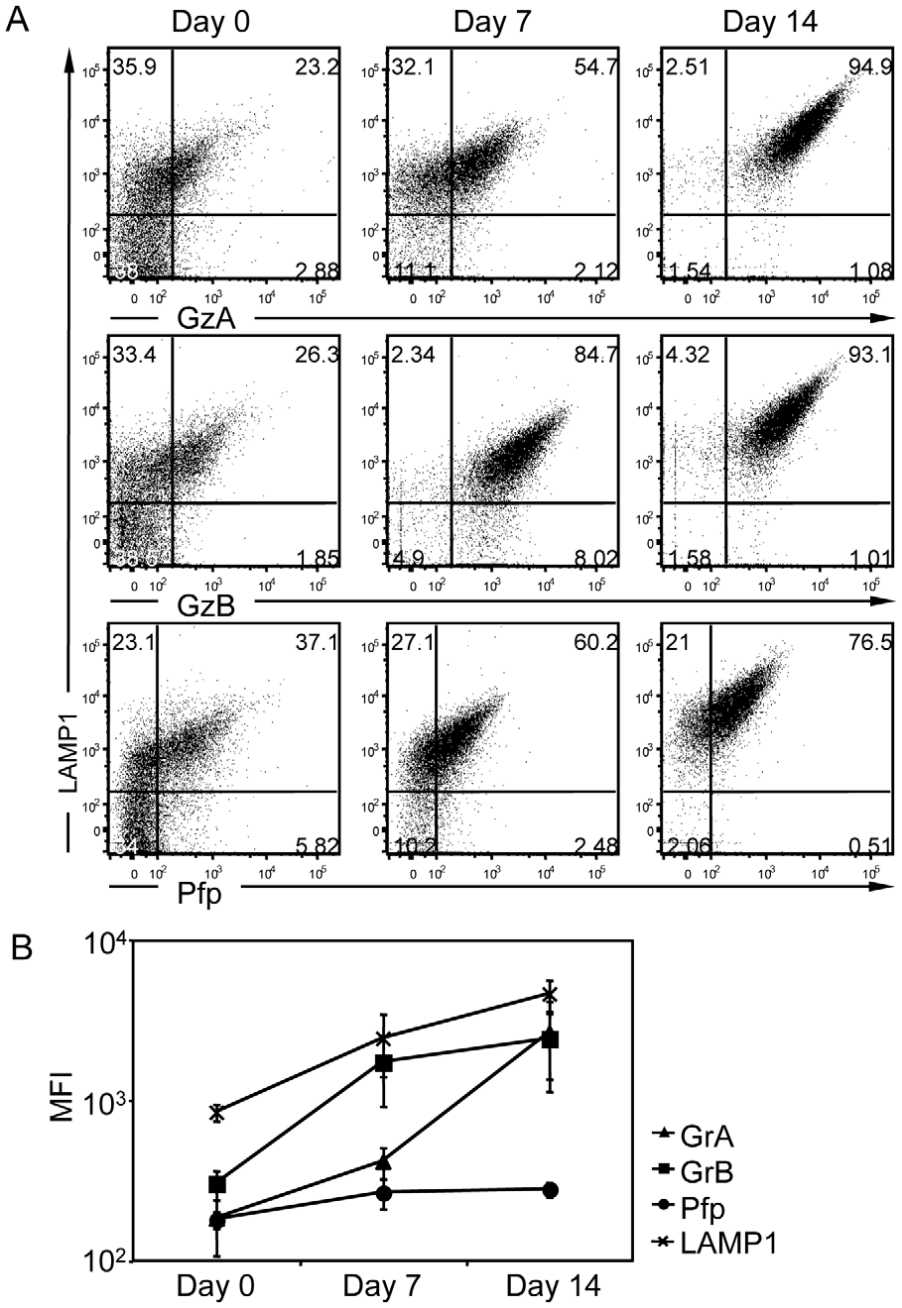
Stepwise maturation of lytic granules along CTL differentiation. (A) Flow cytometric analysis of total vesicular extract from freshly isolated CD8^+^ T cells and at day 7 and 14 after activation. Samples were fixed with 0.2% PFA in the presence of 0.1% picric acid and permeabilized with 0.2% Tween-20 prior to staining with anti-LAMP1, anti-GrA, anti-GrB anti-Pfp and anti-Tia-1 mAbs. The data are from one representative donor out of 3 analyzed in a similar way. (B) The mean fluorescence intensity (MFI) of GrA, GrB and Pfp for CD8^+^ cells at days 0, 7 and 14 are shown (n = 3).

To examine the lytic molecule load of individual lytic granules along CTL differentiation, vesicular extracts purified from CD8^+^ T cells at day 0, 7 and 14 of stimulation were stained with specific antibodies against GrA, GrB, Pfp, LAMP1 and the T-cell-restricted intracellular antigen-1 (Tia-1), a lytic granule-associated RNA binding protein, also associated to stress granules and P-bodies [23]–[25]. At day 0, approximately 60% of the vesicles from CD8^+^ T cells expressed LAMP1 (**Fig. 5A**). A large fraction of these LAMP1^+^ vesicles failed to express the lytic molecules indicating that undifferentiated CTL contain conventional lysosomes. Another fraction of the LAMP1^+^ vesicles expressed high Pfp and GrA/GrB levels. They most probably correspond to lytic granules originating from the minor fraction of differentiated CTL detected at day 0 (**Fig. 1**). At day 7 following stimulation, the proportion of isolated vesicles expressing LAMP1 increased to approximately 85%. The expression levels of the lytic molecules increased substantially within the LAMP1 positive vesicles (**Fig. 5A–B**). The isolated lytic granules harbored a relatively homogeneous lytic molecule load. At this intermediate stage of CTL differentiation, GrB was already expressed at high levels within lytic granules, while GrA and Pfp were expressed at lower levels. At day 14 following stimulation, a further increase in LAMP1^+^ vesicles was observed, reaching >95% of the isolated vesicles. The load in GrB and Pfp increased slightly as compared to day 7 (**Fig. 5A–B**). More remarkably, the load in GrA increased substantially from day 7 to day 14 (**Fig. 5A–B**). In an attempt to further discriminate lytic granules at different stages of their biogenesis, we analyzed the expression of Tia-1. This molecule appeared to be co-expressed with LAMP1 in most of the analyzed vesicles all along CTL differentiation (**Fig. S4**). Interestingly, the lytic molecules GrA, GrB and Pfp appeared only in the vesicles expressing the highest amount of Tia-1 (**Fig. S4**). Together, these data strongly suggest that, during CTL differentiation, the biogenesis of lytic granules relies on the stepwise loading of GrB, Pfp and GrA into LAMP1^+^/Tia-1^high^ lysosomes, leading to the generation of a relatively homogeneous population of lytic granules co-expressing all 3 lytic molecules.

### Quantitative assessment of Rab27a recruitment on lytic granules following activation

**Figure 6:**
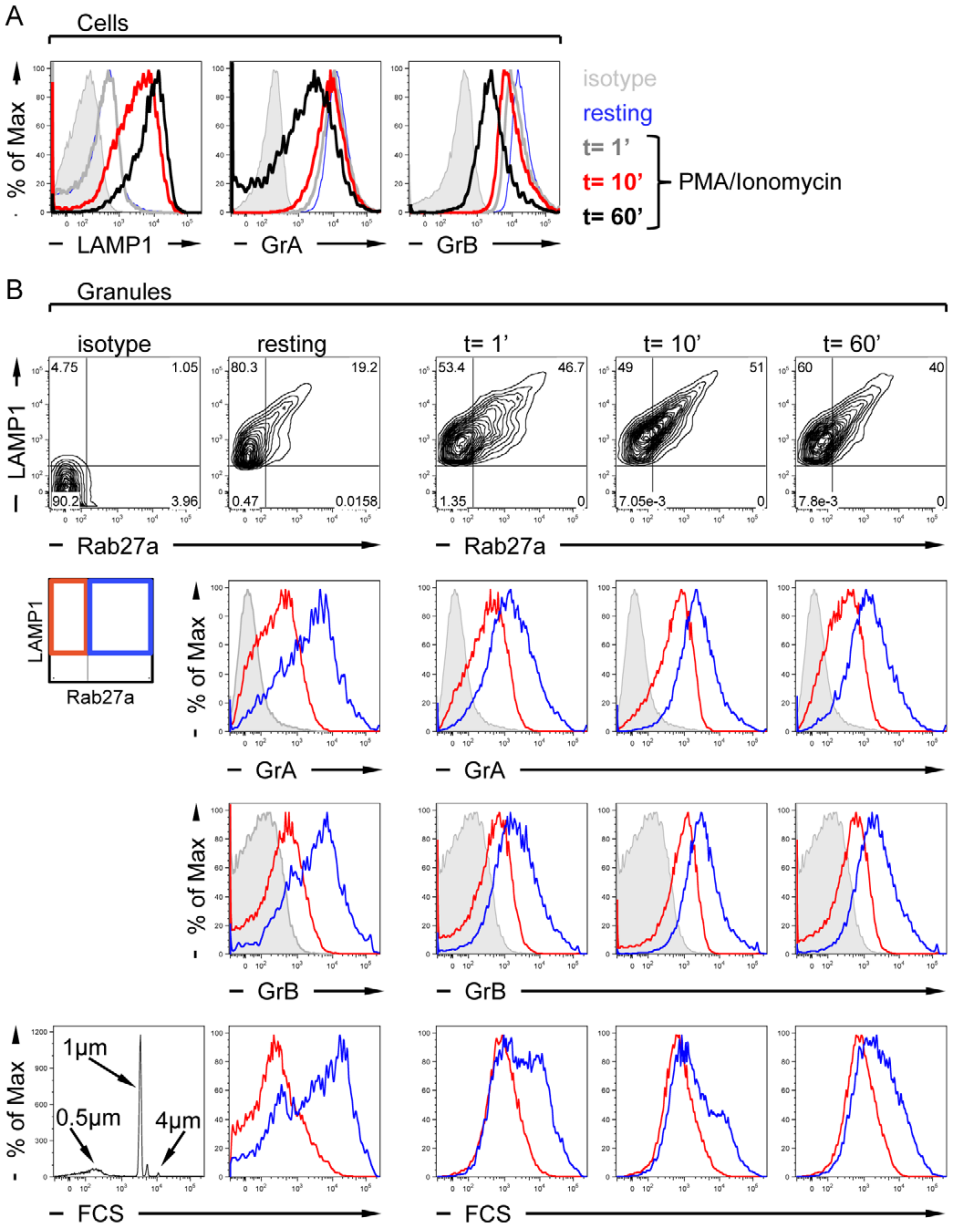
Rapid Rab27a association to lytic granules following CTL stimulation. (A) Cell surface LAMP1 and intracellular GrA and GrB stainings in differentiated CTL either prior (resting) or after PMA/ionomycin stimulation (1, 10 and 60 min). (B) LAMP1^+^ vesicles were analyzed for the presence of Rab27a (first raw). LAMP1^+^/Rab27a^−^ (red) and LAMP1^+^/Rab27a^+^ (blue) vesicles were then compared for their content in GrA (second raw) and GrB (third raw), as well as for their size measured as FCS (fourth raw, including size standard bead calibration). The data are from one representative experiment out of four.

We then evaluated to which extend and with which kinetics lytic granule composition may change upon activation of differentiated CTL. For that purpose, CTL were treated with PMA/ionomycin to trigger calcium-dependent lytic granule exocytosis. As an indication of lytic granule exocytosis, LAMP1 expression at the cell surface started to increase after 10 min of PMA/ionomycin stimulation and further increased after 60 min (**Fig. 6A**). In parallel, the intracellular content in both GrA and GrB clearly decreased following 60min after treatment, as expected. In the lytic granules recovered from the CTL, the presence of Rab27a, a molecule required for lytic granule docking to the plasma membrane, was then monitored. Unexpectedly, prior to stimulation of resting differentiated CTL, a significant proportion (close to 20%) of the lytic granules identified as LAMP1^+^ vesicles was already equipped with Rab27a (**Fig. 6B**). Rab27a appeared to be associated with those lytic granules displaying the highest content in LAMP1. Rapidly after PMA/ionomycin stimulation (1 and 10 min), the proportion of lytic granules associated with Rab27a strongly increased, while it decayed slightly after 60 min. These data suggest that although an important proportion of lytic granules acquires Rab27a rapidly following stimulation, these lytic granules were not all secreted even in the context of a massive exocytic process. We then investigated whether Rab27a^+^ lytic granules present in both resting and activated cells could be distinguished from Rab27a^−^ lytic granules on the basis of lytic molecule content and size. Clearly, the subset of Rab27a^+^ lytic granules harbored an elevated content in GrA and GrB and a much larger size than the Rab27a^−^ counterpart (**Fig. 6B**). Rapidly upon PMA/ionomycin stimulation, the pool of Rab27a^+^ lytic granules remaining within the cells appeared to become less rich in lytic molecules and to become smaller as compared to the initial pool, presumably because of the preferential docking and exocytosis of the richest and largest lytic granules of the Rab27a^+^ pool. Together, these data indicate that the lytic granules with the highest lytic molecule load are preferentially associated with the docking molecule Rab27a. However, not all Rab27a^+^ lytic granules are secreted upon activation, indicating that activated CTL spare a stock of mature lytic granules.

## Discussion

Despite the biological significance of lytic granules as key organelles delivered by CTL and NK cells to lyse infected or malignant host cells, the composition of the lytic granule population during its biogenesis and secretion has previously only been partly characterized. Here, we report a novel approach based on flow cytometry to study the expression levels of membrane-associated molecules and intra-granular lytic molecules in large numbers of lytic granules. This approach was applied to primary human CTL at different stages of differentiation and activation, to precisely measure load in lytic molecules and association with the docking molecule Rab27a.

Out of the crude vesicular extract of CTL, lytic granules were identified based on the presence of both membrane-associated (LAMP1 and Tia-1) and intra-granular molecules (GrA, GrB and Pfp). Differentiated CTL contained a relatively homogeneous pool of lytic granules co-expressing all the above-mentioned molecules. This apparent homogeneity is somewhat in contrast with the analysis of EM pictures showing that lytic granules harbor heterogeneous morphological appearances, as previously reported in murine T cells and human clones [7], [12], [26]. The analysis of lytic granule diameter size distribution by flow cytometry assisted by size standard beads also failed to discriminate lytic granule subsets since most lytic granules distributed in a broad but continuous fashion from a few hundreds of a µm to a few µm, as previously noted [8]. Therefore, our flow cytometry analysis indicates that the lytic granule population of differentiated human CTL is made of individual granules that distribute in a continuous fashion in terms of size and lytic molecule load.

By measuring the load of individual lytic molecules within lytic granules during the differentiation of CTL, we gained insight into the biogenesis of these organelles. In the crude vesicular extract from freshly isolated CD8^+^ T cells, LAMP1^+^ vesicles not containing lytic molecules were detected, indicating that CD8^+^ T cells isolated from human blood harbor conventional lysosomes or secretory lysosomes lacking lytic molecules. In addition to lysosomes, freshly isolated CD8^+^ T cells also contained lytic granules. These lytic granules probably derived from the subset of differentiated CTL present within the blood samples, as detected by the expression of lytic molecules.

Upon stimulation with anti-CD3/CD28 Ab-coated beads, CD8^+^ T cells displayed a differentiated phenotype with all cells co-expressing the lytic molecules GrA, GrB and Pfp. Analysis of vesicular extracts indicates that these cells were devoid of conventional lysosomes suggesting that along CTL differentiation lysosomes were replaced by lytic granules or that they matured into lytic granules. Notably, the proportion of the LAMP1^+^ vesicles within the vesicular extract increased with stimulation time as compared to freshly isolated CD8^+^ T cells, thereby indicating that differentiating CTL restrict an important part of their vesicular equipment to lysosome-type vesicles. This is in accordance with the increase of LAMP1 and additional lysosomal proteins after lymphocyte activation [6].

At the cellular level, lytic molecules are acquired in a stepwise manner during CTL differentiation [27], [28]. Indeed, different transcriptional programs are known to regulate lytic molecule expression during CTL differentiation [29]. In our lytic granule analysis, GrB was the first of the 3 lytic molecules tested to saturate the LAMP1^+^/Tia-1^high^ compartment following stimulation with anti-CD3/CD28 Ab-coated beads. This was the case at day 7 of stimulation when CD8^+^ T cells had reached an intermediate stage of differentiation, as assessed previously [30]. The precocity of GrB loading into lytic granules is in accordance with the parallel analysis on whole cells showing homogeneously high expression of GrB at day 7 of stimulation. GrA and Pfp targeting into the LAMP1^+^/Tia-1^high^ compartment appeared to be delayed as compared to that of GrB. This delay is probably due to the upregulation of mRNA transcripts taking place later during differentiation [31] or could be linked to the presence of post-transcriptional regulatory mechanisms [32]. Our data also indicate that during CTL differentiation the size distribution of the LAMP1^+^ vesicle pool remained relatively constant (**Fig. S5**). The recovered lytic granules appeared homogeneous in their load in the different molecules studied. This homogeneity was confirmed when considering dot plots showing the staining for the 3 molecules in different combinations (**Fig. S6**). These data indicate that CTL differentiation is accompanied by the stepwise maturation of a relatively homogeneous pool of lytic granules that concentrate the different lytic molecules.

By measuring the association of lytic granules from differentiated CTL to the docking molecule Rab27a [19], [33] following PMA/ionomycin activation, we gained insight into the dynamics by which lytic granules get mobilized for secretion. Although lytic granules are considered not to be intrinsically prone to secretion, we reproducibly measured that a fraction of the lytic granules collected from differentiated CTL was already associated to Rab27a prior to activation. It will be of interest to further investigate by which mechanisms CTL control the rate of association of lytic granules to Rab27a. Such an association suggests that differentiated CTL contain a set of granules prone to rapid secretion and that Rab27a is not the only rate-limiting step of secretion. In agreement, the acquisition of additional molecules such as Munc14-3, via the fusion with endosomal/exocytic vesicles, is required as a final step allowing membrane fusion and subsequent exocytosis [18]–[20]. In addition to the regulation of docking, Rab27a may also have additional functions such as the control of intracellular lytic granule motility as recently described [34].

Beyond the apparently homogeneous lytic molecule load of mature CTL lytic granules, Rab27a expression defined two distinct lytic granule subsets in terms of lytic molecule load and size. This indicates that lytic granules coexist in CTL under distinct maturation stages, although such stages cannot be discriminated with the sole analysis of lytic molecule load, probably because of a continuous distribution of this load among individual granules. The presumably more mature Rab27a^+^ lytic granules were associated with the highest lytic molecule load. The fact that Rab27a^+^ lytic granules were much larger than the Rab27a^−^ lytic granules supports the notion that lytic granules fuse to each other and with endosomal structures during maturation, along the steps preceding exocytosis [20]. Very rapidly upon stimulation, the proportion of lytic granules associated to Rab27a increased. In agreement with previous studies, not all lytic granules recruited Rab27a [15]. It is possible that other granules get associated with alternative docking mediators [35] or that the limited Rab27a recruitment is a mechanism for differentiated CTL to hold part of their lytic granules.

Our study indeed highlights the fact that CTL do not deplete their lytic granule stock, even under conditions of strong stimulation. This may result from both a limited Rab27a recruitment and from a limited secretion of the lytic granules associated to Rab27a. Sparing a pool of lytic granules is probably key to execute multiple and sequential killing [36]. Additionally, since differentiated CTL appear not to contain conventional lysosomes, the house-keeping degradative functions are most probably ensured by non-secreted lytic granules [7].

In conclusion, our novel approach proves to be a powerful tool for analyzing lytic granule composition during CTL differentiation and activation. This methodology opens new possibilities to better elucidate, in complement with other approaches, the molecular regulation of lytic granule biogenesis and trafficking in both health and pathological settings.

## Materials and Methods

### Isolation, differentiation and activation of primary human CD8^+^ T lymphocytes

Peripheral blood mononuclear cells (PBMC) were isolated from buffy coats of healthy donors obtained through the Etablissement Français du Sang (EFS Midi-Pyrénées, Purpan University Hospital, Toulouse, France). Blood samples were collected and processed following standard ethical procedures (Helsinki protocol), after obtaining written informed consent from each donor and approval for this study by the local ethical committee (Comité de Protection des Personnes Sud-Ouest et Outremer II). CD8^+^ T cells were purified using the CD8 MicroBead Kit (Miltenyi Biotec), according to the manufacturer's instructions. CD8^+^ T cells were stimulated and expanded with anti-CD3/anti-CD28 T-Cell Expander Dynabeads™ (Invitrogen) in RPMI 1640 medium supplemented with 10% FCS, 2 mM L-Glutamine, 1 mM Sodium Pyruvate, 10 mM Hepes, 50 U/ml Penicillin, 50 µg/ml Streptomycin (all from Invitrogen), 50 µM b-Mercaptoethanol, 150 U/ml Interleukin-2 (Chiron/Proleukin), 10 ng/ml Interleukin-7 (R&D System) and 5 ng/ml Interleukin-15 (R&D System). Cultures were maintained at 37°C and 95% air, 5% CO_2_ in an humidified incubator. After 14 to 20 days in culture, differentiated CTL (2.0×10^7^ cells) were activated for 0, 10 or 60 min with 0.1 µg/ml Phorbol Myristate Acetate (PMA) and 1 µg/ml Ionomycin (both from Sigma).

### Monoclonal antibodies used for flow cytometry analysis

The following murine mAb were used to stain cells and/or isolated vesicles for the purpose of flow cytometry analysis: CD3-PE (BD, clone RPA-T4), CD8-AmCyan (BD, clone SK1), CD8-PeCy7 (BD, clone RPA-T8), LAMP1-PerCP-Cy5.5 (Biolegend, clone H4A3), LAMP1-PE (BD, clone H4A3), GrA-Pacific Blue (Biolegend, clone CB9), GrB-FITC (BD, clone GB11), GrB-Alexa700 (BD, clone GB11), Pfp-FITC (Diaclone, clone B-D48), Pfp-Alexa647 (Biolegend, clone dG9), Tia-1-PE (Beckman Coulter, clone 2G9), Rab27a (Abnova, clone 1G7) followed by Alexa633- or PE-labeled goat anti mouse Ab (Molecular Probes or Southern Biotech, respectively). IgG isotypes were used as staining controls for each Ab isotype/fluorochrome combination.

### Cell immunolabeling

PBMC and CD8^+^ T cells (1×10^6^) harvested at different days after the stimulation were first stained at 4°C for 30 min with specific mAbs against the CD8 molecule. The cells were then washed twice with 3% BSA (Sigma), 10 mM Hepes in PBS. To determinate the intracellular expression of GrA, GrB and Pfp, cells were fixed with 4% PFA (Sigma) in PBS at 4°C for 10 min and then permeabilized with 3% BSA, 10 mM Hepes, 0.1% Saponin (Sigma) in PBS at 4°C for 20 min. Staining for intracellular molecules were performed at 4°C for 30 min. Following 2 washing steps with permeabilizing buffer, samples were reconstituted with 0.25 ml of 3% BSA, 10 mM Hepes, 4 mM EGTA (Sigma) in PBS.

### Lytic granule isolation

Granule isolation was performed following a published protocol [37] with minor modifications. Briefly, CD8^+^ T cells were washed three times with ice cold PBS and reconstituted in 2×10^7^cells/ml of relaxation buffer: 130 mM KCl; 5 mM NaCl; 2 mM MgCl2; 1 mM disodium-ATP/10 mM Hepes pH 7.4, containing 1 mM PMSF and protease inhibitors cocktail (Sigma). Cells were disrupted by N_2_ cavitation at 400p.s.i and the cell suspension was collected in the presence of 4 mM EGTA. The cell lysate was separated into a post-nuclear supernatant (SN1) and a pellet (P1) by centrifugation at 800×g for 10 min at 4°C. SN1 was submitted to another centrifugation step at 20 000×g for 30 min at 4°C which resulted in a cytosolic supernatant (SN2) and a total vesicular extract enriched in the pellet P2.

### Fixation and immunolabeling of isolated lytic granules

Three protocols were tested in parallel to optimize the labeling of lytic granule surface and intra-granular antigens. Equal aliquots of the P2 fraction were treated in each of the following ways: 1- no treated; 2- permeabilized with 0.25% Tween-20; 3- fixed with 0.2% PFA and permeabilized with 0.25% Tween-20; 4- fixed with 2% PFA, 0.1% Picric Acid and permeabilized with 0.25% Tween-20. Stainings with specific antibodies were performed over-night at 4°C in 0.22 µm-filtered lytic granule buffer: 0.5% BSA, 10 mM Hepes pH7.4, 4 mM EGTA. All solutions were prepared in PBS. Lytic granules were washed twice and reconstituted with lytic granule buffer before FACS analysis. Centrifugation steps were at 20 000 g, 4°C for 30 min.

### Flow cytometry

The stained cells were submitted to parametric flow cytometry analysis using a LSRII flow cytometer (BD Biosciences) equipped with four lasers (355, 405, 488, 633 nm). Compensation was performed for each experiment using CompBeads compensation particles (BD Biosciences). A minimum of 20 000 lymphocyte events were collected for analysis. The stained granules were analyzed using the same instrument but previous calibration with size standards microbeads (Sigma) of 0.5, 1.0 and 4.0 µm was needed using logarithmic amplifiers. Instrument FSC and SSC thresholds were set to 200 to subtract the noise and PMT voltages were balanced to resolve the different standard beads. A minimum of 50 000 events were collected for analysis. Data were analyzed using the FlowJo software (Tree Star).

### Western blot

Cells were lysed in ice-cold lysis buffer, 50 mM Tris-Cl (pH 7.5), 150 mM NaCl, 5 mM MgCl_2_, 1% Nonidet P-40, 1 mM DTT, 5% Glycerol. Fraction (P2) enriched in lytic granules and supernatants (SN1, and SN2) were boiled in Laemmli buffer for 5 min. Proteins corresponding to 10% of total homogenate were separated on a 12% SDS-PAGE and transferred to nitrocellulose membranes (Amersham) which were blocked in 3% BSA in PBS, then incubated with anti-GrB mAb (Dako Cytomation, clone GrB-7) and finally with HRP-conjugated goat anti-mouse IgG secondary Ab (eBioscience). Incubations were in PBST containing 3% BSA for 1 h at room temperature. Protein bands were visualized using a Supersignal West Pico Chemiluminescence Substrate Kit (Thermo Scientific).

### Transmission electron microscopy

The P2 fraction, isolated from stimulated CTL after 17 days in culture, was fixed in 2% glutaraldehyde in 0.1 M Sorensen phosphate buffer (pH 7.4) for 4 h at 4°C, resuspended in 2% low melting point agarose, washed over-night in 0.2 M phosphate buffer and then post-fixed for 1 h at room temperature with 1% osmium tetroxide in 250 mM sucrose and 0.1 M phosphate buffer. The sample was then dehydrated in a series of graded ethanol solutions and embedded in an Epon-araldite resin (Embed 812-Araldite 502, Electron Microscopy Sciences). Finally, the sample was sliced into 70-nm thick sections (Ultracut Reichert Jung) and mounted on 100-mesh collodion-coated copper grids prior to staining with 3% uranyl acetate in 50% ethanol and Reynold's lead citrate. Examinations were carried out on a transmission Hitachi HU12A electron microscope at an accelerating voltage of 75 kV.

## Supporting Information

Figure S1 **Co-expression of lytic molecules during CD8^+^ T cells differentiation.** Expression of GrA, GrB and Pfp in purified CD8^+^ T cells at days 0, 7 and 17 after stimulation with anti-CD3/CD28 mAb-coated beads in the presence of IL-2, Il-7 and IL-15. Cells were stained with anti-GrA, anti-GrB and anti-Pfp mAbs and analyzed by flow cytometry. The co-expression levels of the 3 lytic molecules are plotted as dot plots showing all combinations. The values in each plot show the frequency of each subset in the cell population. The data are from one representative donor out of 3 analyzed in a similar way.
(TIF)

Figure S2 **Size distribution of CTL vesicles.** Relative distribution of vesicles recovered from mature CTL in the G1 to G4 size gates, elaborated with standard beads. Data represent the mean and SD of 7 vesicular extracts obtained from differentiated CTL.
(TIF)

Figure S3 **Distinction of vesicles isolated from B cells and CD8^+^ T cells.** Flow cytometry analysis of LAMP1 and GrA expression in total vesicular extracts from B cells (JY cell line) and CD8^+^ T cells. The third panel shows the distinction of B cell and CD8^+^ T cell vesicles following their mixing prior to fixation/permeabilization and staining. The data are from one representative experiment out of 3.
(TIF)

Figure S4 **Lytic granules express high Tia-1 levels.** Expression of Tia-1, LAMP1 and the lytic molecules GrA, GrB and Pfp measured by flow cytometry in purified CD8^+^ T cells at days 0, 7 and 17 after stimulation with anti-CD3/CD28 mAb-coated beads in the presence of IL-2, Il-7 and IL-15. The values in each plot show the frequency of each subset in the cell population.
(TIF)

Figure S5 **Size distribution of LAMP1^+^ vesicles during CTL differentiation.** Flow cytometry analysis of the physical parameters (SSC-A, proportional to granulometry and FSC-A, proportional to size) of vesicles from CD8^+^ T cells at different stages of differentiation. Vesicles were stained for LAMP1 and only LAMP1^+^ vesicles were considered in the analysis. Numbers indicate the percentage of vesicles distributing in the G1 to G4 size gates elaborated with standard beads. The data are from one representative donor out of 3 analyzed in a similar way.
(TIF)

Figure S6 **Co-expression of lytic molecules during lytic granule biogenesis.** Expression of GrA, GrB and Pfp in vesicles purified from CD8^+^ T cells at days 0, 7 and 17 after stimulation with anti-CD3/CD28 mAb-coated beads in the presence of IL-2, Il-7 and IL-15. Isolated vesicles were fixed/permeabilized and stained with anti-GrA, anti-GrB and anti-Pfp mAbs and analyzed by flow cytometry. The co-expression levels of the 3 lytic molecules are plotted as dot plots showing all combinations. The values in each plot show the frequency of each subset in the vesicle population. The data are from one representative donor out of 3 analyzed in a similar way.
(TIF)
