# Supplementary figures and images for: Stepwise Maturation of Lytic Granules during Differentiation and Activation of Human CD8+ T Lymphocytes
Source: PLoS One. 2011 Nov 4;6(11):e27057. doi: 10.1371/journal.pone.0027057 (PMC3208563; doi:10.1371/journal.pone.0027057)

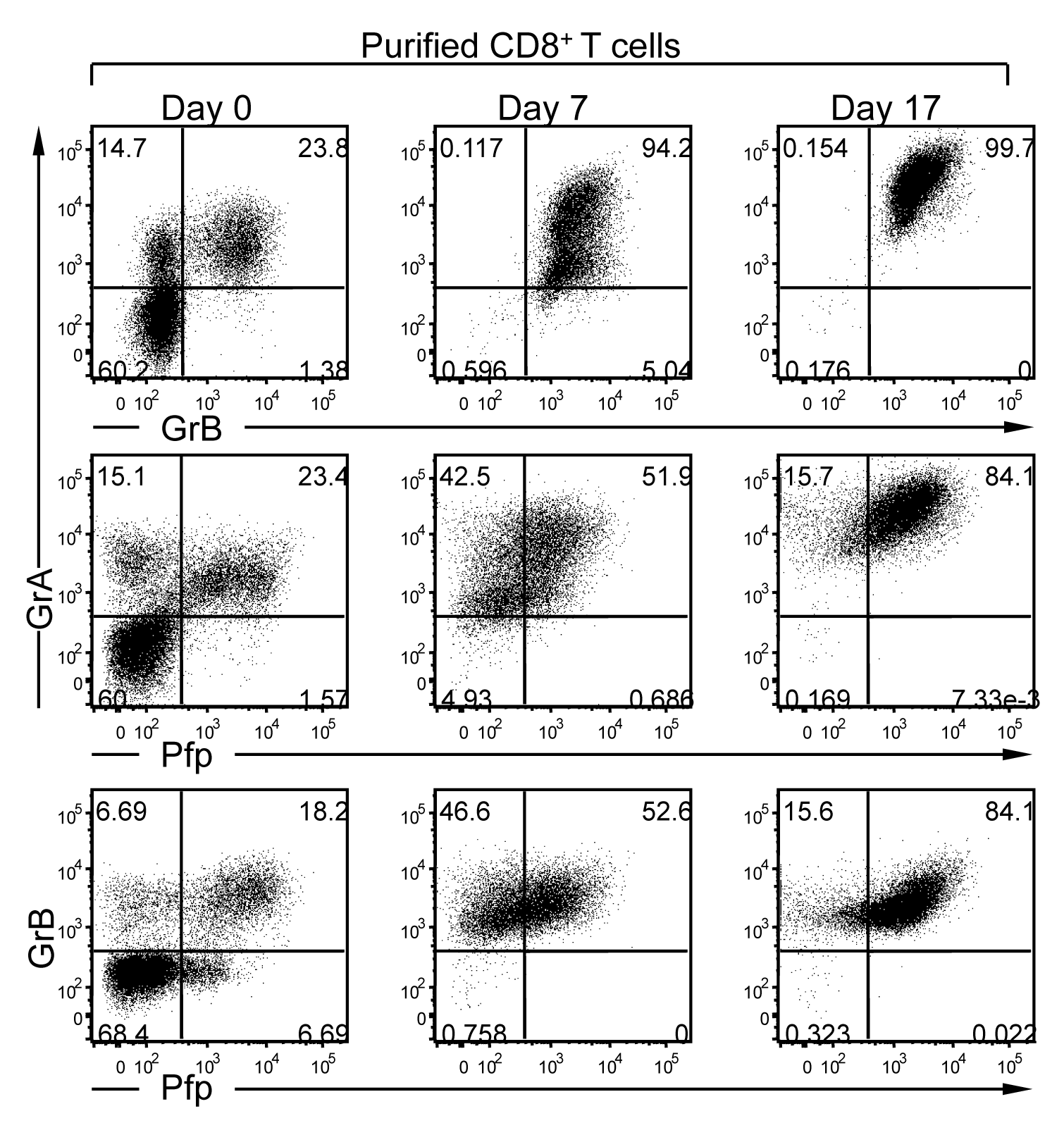

Supplement: Figure S1 — Co-expression of lytic molecules during CD8+ T cells differentiation. Expression of GrA, GrB and Pfp in purified CD8+ T cells at days 0, 7 and 17 after stimulation with anti-CD3/CD28 mAb-coated beads in the presence of IL-2, Il-7 and IL-15. Cells were stained with anti-GrA, anti-GrB and anti-Pfp mAbs and analyzed by flow cytometry. The co-expression levels of the 3 lytic molecules are plotted as dot plots showing all combinations. The values in each plot show the frequency of each subset in the cell population. The data are from one representative donor out of 3 analyzed in a similar way. (TIF) [file pone.0027057.s001.tif]

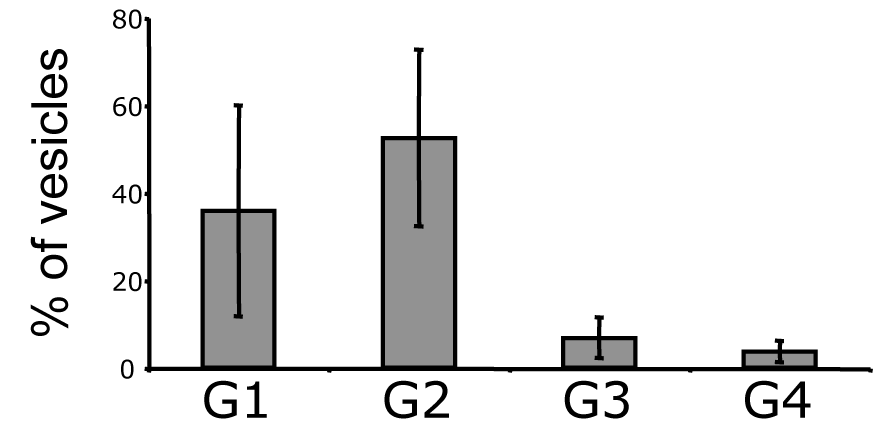

Supplement: Figure S2 — Size distribution of CTL vesicles. Relative distribution of vesicles recovered from mature CTL in the G1 to G4 size gates, elaborated with standard beads. Data represent the mean and SD of 7 vesicular extracts obtained from differentiated CTL. (TIF) [file pone.0027057.s002.tif]

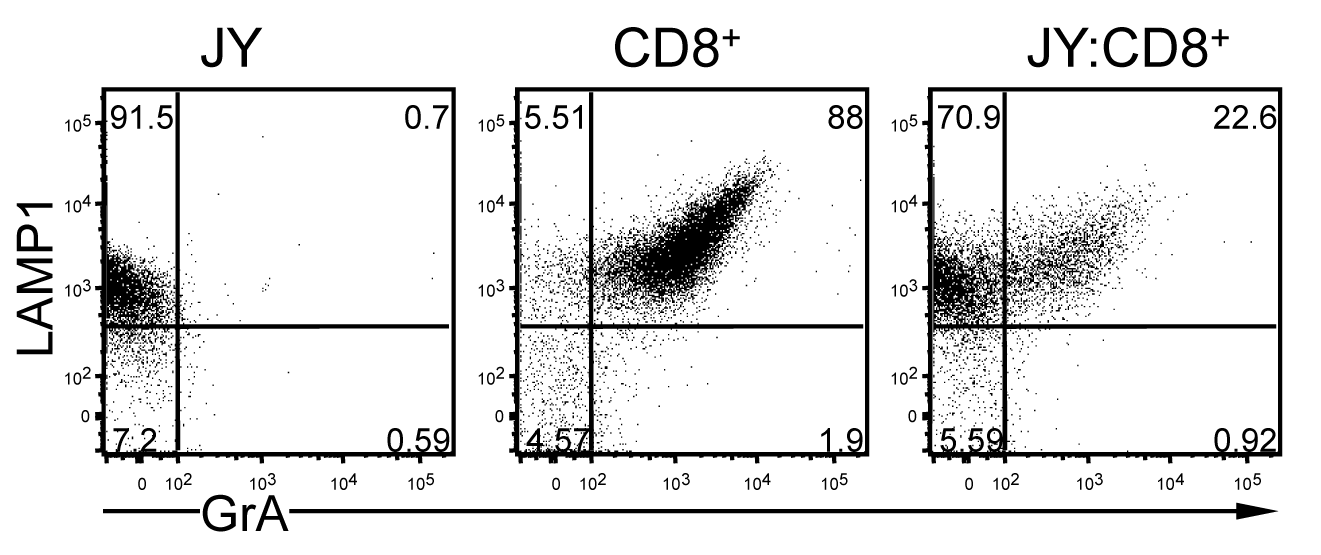

Supplement: Figure S3 — Distinction of vesicles isolated from B cells and CD8+ T cells. Flow cytometry analysis of LAMP1 and GrA expression in total vesicular extracts from B cells (JY cell line) and CD8+ T cells. The third panel shows the distinction of B cell and CD8+ T cell vesicles following their mixing prior to fixation/permeabilization and staining. The data are from one representative experiment out of 3. (TIF) [file pone.0027057.s003.tif]

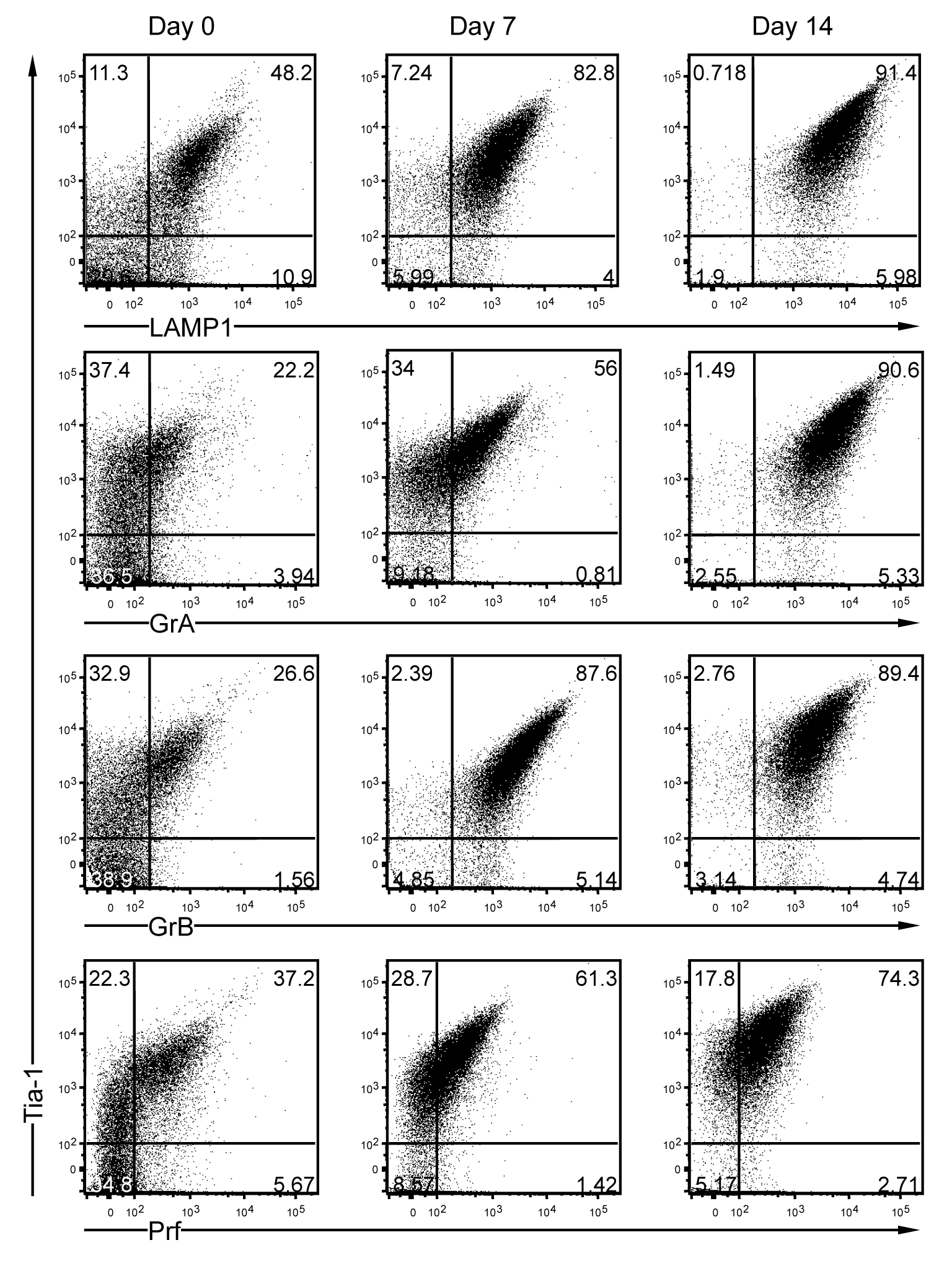

Supplement: Figure S4 — Lytic granules express high Tia-1 levels. Expression of Tia-1, LAMP1 and the lytic molecules GrA, GrB and Pfp measured by flow cytometry in purified CD8+ T cells at days 0, 7 and 17 after stimulation with anti-CD3/CD28 mAb-coated beads in the presence of IL-2, Il-7 and IL-15. The values in each plot show the frequency of each subset in the cell population. (TIF) [file pone.0027057.s004.tif]

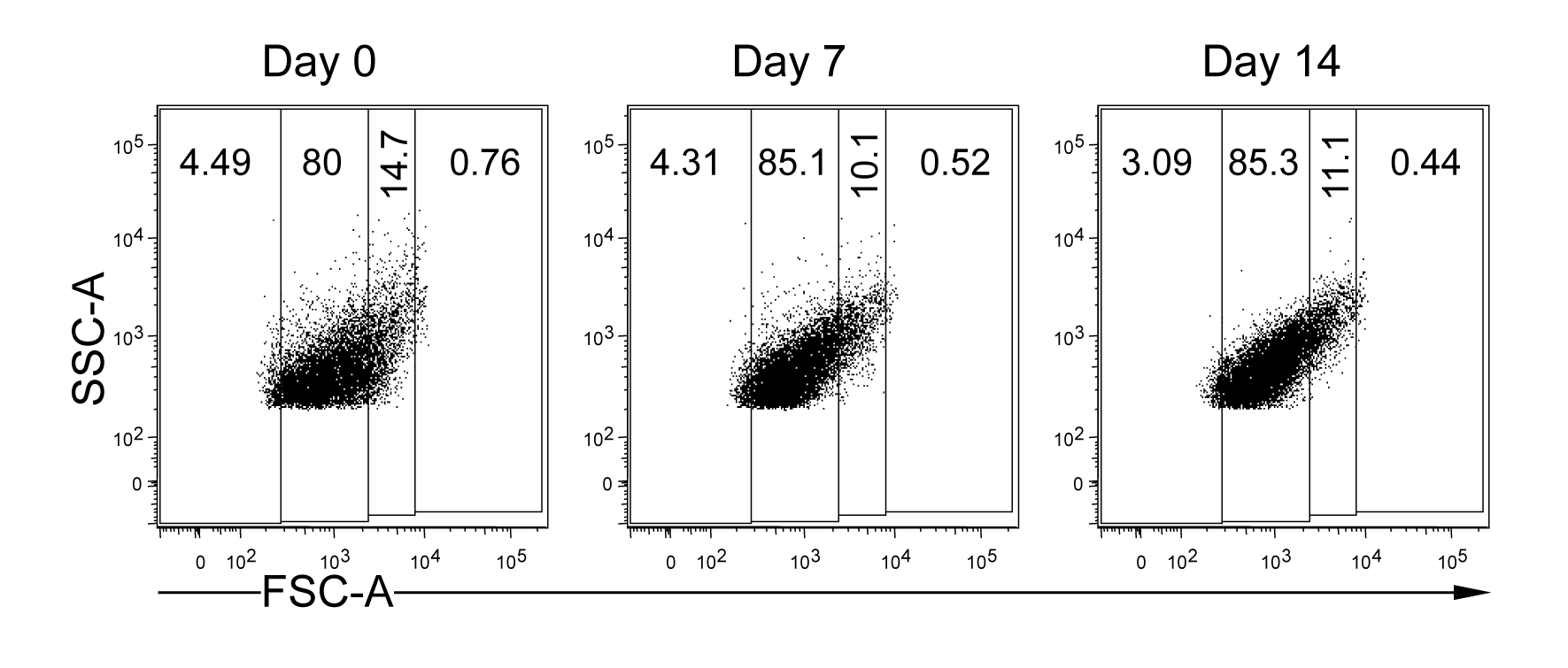

Supplement: Figure S5 — Size distribution of LAMP1+ vesicles during CTL differentiation. Flow cytometry analysis of the physical parameters (SSC-A, proportional to granulometry and FSC-A, proportional to size) of vesicles from CD8+ T cells at different stages of differentiation. Vesicles were stained for LAMP1 and only LAMP1+ vesicles were considered in the analysis. Numbers indicate the percentage of vesicles distributing in the G1 to G4 size gates elaborated with standard beads. The data are from one representative donor out of 3 analyzed in a similar way. (TIF) [file pone.0027057.s005.tif]

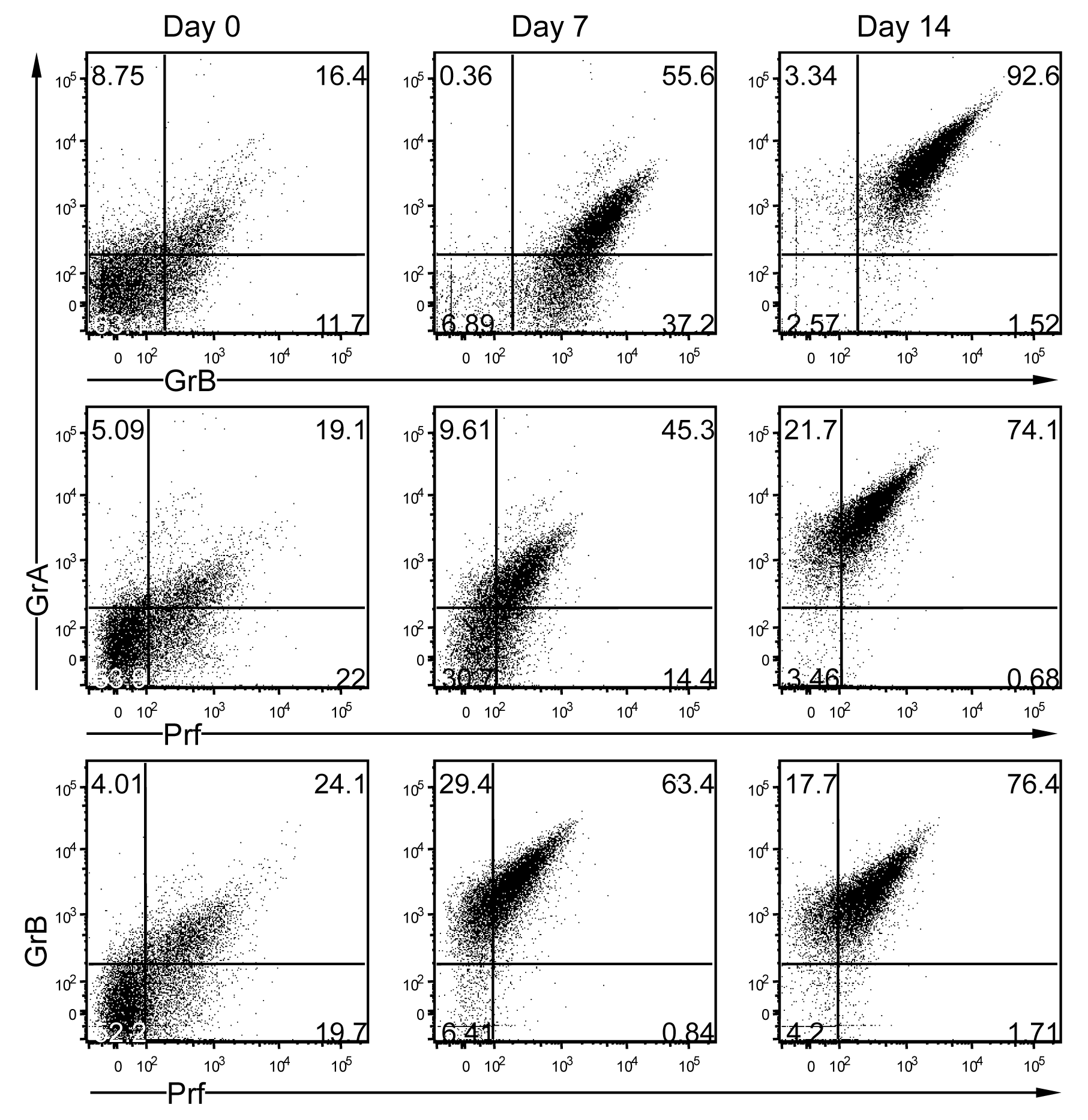

Supplement: Figure S6 — Co-expression of lytic molecules during lytic granule biogenesis. Expression of GrA, GrB and Pfp in vesicles purified from CD8+ T cells at days 0, 7 and 17 after stimulation with anti-CD3/CD28 mAb-coated beads in the presence of IL-2, Il-7 and IL-15. Isolated vesicles were fixed/permeabilized and stained with anti-GrA, anti-GrB and anti-Pfp mAbs and analyzed by flow cytometry. The co-expression levels of the 3 lytic molecules are plotted as dot plots showing all combinations. The values in each plot show the frequency of each subset in the vesicle population. The data are from one representative donor out of 3 analyzed in a similar way. (TIF) [file pone.0027057.s006.tif]
